# Myocardial Blood Flow and Flow Reserve in Patients With Acute Myocardial Infarction and Obstructive and Non-Obstructive Coronary Arteries: CZT SPECT Study

**DOI:** 10.3389/fnume.2022.935539

**Published:** 2022-07-06

**Authors:** Konstantin V. Zavadovsky, Darya A. Vorobyeva, Olga V. Mochula, Andrew V. Mochula, Alina N. Maltseva, Andrew E. Bayev, Marina O. Gulya, Alessia Gimelli, Vyacheslav V. Ryabov

**Affiliations:** ^1^Tomsk National Research Medical Centre, Cardiology Research Institute, Russian Academy of Sciences, Moscow, Russia; ^2^Fondazione Toscana, CNR Gabriele Monasterio, Pisa, Italy

**Keywords:** acute myocardial infarction, myocardial perfusion imaging, myocardial blood flow (MBF), cardiac troponin I (cTnI), cardiac magnetic resonance (CMR) imaging

## Abstract

**Background:**

To assess single-photon emission computed tomography cadmium-zinc-telluride (SPECT CZT)-derived myocardial blood flow (MBF) flow reserve (MFR) and flow difference (FD) in patients with acute myocardial infarction (AMI) and to compare this data with serum cardiac troponin and cardiac magnetic resonance (CMR) findings.

**Methods:**

A total of 31 patients with AMI underwent invasive coronary angiography (ICA), serial high-sensitivity serum cardiac troponin I (cTnI) measurement, and CZT SPECT with visual and quantitative (MBF, MFR, and FD) perfusion parameters, and contrast-enhanced CMR. All patients with AMI were divided into two groups: (1) with non-obstructive coronary arteries (MINOCA), *n* = 10; (2) with obstructive coronary artery disease (MICAD), *n* = 21.

**Results:**

The values of SSS and SRS were significantly (*p* < 0.01) higher whereas global stress MBF, MFR significantly lower in patients with MICAD as compared to MINOCA – 5.0 (3.0; 5.0) *vs*. 9.0 (5.0; 13.0); 2.0 (1.0; 3.0) *vs*. 6.0 (3.0; 11.0); 2.02 (1.71; 2.37) *vs*. 0.86 (0.72; 1.02) ml/min/g; and 2.61 (2.23; 3.14) *vs*. 1.67 (1.1; 1.9), respectively. Stress MBF correlated with cTnI at 24 h and day 4: ρ = −0.39; *p* = 0.03 and ρ = −0.47; *p* = 0.007, respectively. FD correlated with cTnI at 24 h and day 4: ρ = −0.39; *p* = 0.03 and ρ = −0.46; *p* = 0.009. CMR analysis showed that infarct size, MVO and myocardial edema in patients with MICAD were significantly (< 0.05) higher as compared to MINOCA: 19.4 (10.4; 29.7) *vs*. 1.8 (0.0; 6.9); 0.1 (0.0; 0.7) *vs*. 0.0 (0.0; 0.0) and 19.5 (12.0;30.0) *vs*. 3.0 (0.0; 12.0), respectively. According to vessel-based analysis of CMR data, acute myocardial injury (defined as late gadolinium enhancement and myocardial edema) was observed more frequently in patients with MICAD compared to MINOCA: 34(37%) *vs*. 5(5%) *p* = 0.005, respectively. The values of regional stress MBF, MFR and FD were significantly decreased in LV territories characterized by myocardial injury compared to those without: 0.98 (0.73; 1.79) *vs*. 1.33 (0.94; 2.08) *p* < 0.01, 1.64 (1.0; 2.36) *vs*. 2.0 (1.53; 2.89) *p* < 0.01 and 0.33 (0.05; 0.57) *vs*. 0.56 (0.36; 1.32) *p*> 0.01, respectively.

**Conclusion:**

In patients with AMI, SPECT CZT-derived flow measures were associated with the high-sensitivity troponin I as well as the extent of edema, microvascular obstruction, and infarct size detected by CMR. On the regional level, quantitative SPECT CZT measures were significantly lower in vessel territories characterized by myocardial injury.

## Introduction

Myocardial blood flow (MBF) and myocardial flow reserve (MFR) are well-established quantitative non-invasive measures reflecting coronary pathophysiology in the broad spectrum of cardiac pathology (1). The gold standard of non-invasive MBF and MFR assessment is positron emission tomography (PET) with ^15^O-H_2_O and ^13^N-ammonia. This method showed high accuracy in the diagnosis of functionally significant stenosis (2, 3) in patients with stable obstructive CAD as well as in ischemia without flow-limiting stenosis (4). Moreover, decreased values of MBF and MFR were demonstrated in the territory of infarct-related artery and reference artery as well (5). Further, reduced MFR in non-viable left ventricular (LV) segments in patients with successfully revascularized acute myocardial infarction (AMI) was shown using ^13^N-ammonia PET (6).

However, the low availability and high cost of cardiac PET hinder the wide spread of this method (7). Good agreement between cadmium-zinc-telluride (CZT) single-photon emission computed tomography (SPECT) and PET-derived flow and reserve results were reported in previous studies with ^15^O-H_2_O (8), 13N-ammonia (9, 10) and ^82^Rb (11). Although the diagnostic (12, 13) and prognostic (14) value of CZT SPECT is well studied in patients with stable CAD, the data regarding global and regional MBF and MFR in patients with acute coronary syndrome (ACS) is lacking. Moreover, there is only one study reported the correlation of CZT-derived flow indexes with MR-measured hyperemic MBF and CFR in 30 stable CAD subjects (15). No data is available regarding the association of serial markers of myocardial necrosis measurement, in particular, high-sensitivity serum cardiac troponin I (cTnI) and SPECT CZT derived quantitative indexes of MBF and MFR.

The study of MBF and MFR in patients with AMI may provide additional data to assess the extent and severity of microcirculation impairment due to epicardial artery lesion, subsequence myocardial edema, and microvascular obstruction (MVO) as well as reperfusion injury. It may be of help to triage patients and improve the risk stratification, particularly in subjects with AMI and no obstructive coronary arteries.

For this purpose, this study aimed to assess SPECT CZT-derived myocardial blood flow and flow reserve in patients with AMI and to compare this data with serum cardiac troponin and cardiac magnetic resonance findings.

## Methods

### Study Design

In accordance with the “Cardiac Structure, Function, and Clinical Manifestations in MINOCA” study design (clinicaltrials.gov NCT03572023) all patients with the acute coronary syndrome, submitted to invasive coronary angiography (ICA), with and without obstructive coronary arteries, were enrolled in the study. Patients with obstructive coronary arteries (myocardial infarction with obstructive coronary arteries, MICAD) were enrolled as a control group. Patients with either normal coronaries or no obstructive (<50%) coronary atherosclerosis were identified as myocardial infarction with non-obstructive coronary arteries (MINOCA), while patients with stenosis 50% or higher of one coronary artery were identified as MICAD.

Inclusion criteria were the following: age 18 years and older, ACS who underwent ICA within 24 h of the onset of the disease, high and moderate cardiovascular risk on the Grace scale, and sinus rhythm on ECG. Exclusion criteria were: hemodynamic instability, myocardial inflammatory diseases, storage diseases, moderate-to-severe cardiac valvular disease, atrial fibrillation, previous revascularization, severe comorbidity, severe renal failure (eGFR <30), pacing, claustrophobia, contraindication to adenosine administration.

Rest ECG and high-sensitivity serum cardiac troponin I measurement were performed on admission and after 24 h. Within 2 weeks after ICA, all patients underwent stress/rest SPECT myocardial perfusion scintigraphy (MPS) with quantitative mMBF and MFR assessment as well as cardiac magnetic resonance (CMR) with late gadolinium enhancement (LGE) for the assessment of AMI.

All patients were followed up for 1 year. The primary endpoint was major adverse cardiovascular events (MACE), including all-cause of death, myocardial infarction, and revascularization.

The study was approved by the Local Ethical Committee and conformed to the Declaration of Helsinki on human research. Written informed consent was obtained from every patient after an explanation of the protocol, its aims, and potential risks.

### Serum Cardiac Biomarkers

High-sensitivity serum cardiac troponin I was measured 3 times (in 24 h, 4 days, and 7 days after the admission). cTnI levels were determined by Immunoassay Systems Access (Beckman Coulter, Brea, CA, USA). The upper limit of the norm was conventionally taken from the 99th percentile from the upper reference level (cTnI laboratory reference cut-off for normalcy: <0.04 ng/ml).

### Invasive Coronary Angiography

All patients underwent quantitative coronary arteriography on an Axiom Artis coronary angiography system (Siemens; Erlangen, Germany). Coronary angiography in all patients was performed using a 5F Judkins-type catheter through the femoral approach. All patients received oral aspirin, an intravenous bolus injection of 5,000 IU of heparin, and intracoronary isosorbide dinitrate (2 mg) before angiography. All coronary artery stenoses were quantitatively assessed using dedicated software by two experienced readers (AEB and SIV). Coronary artery stenosis ≥50% in major epicardial coronary arteries and in the left main coronary artery was considered significant. The degree of perfusion was evaluated according to thrombolysis in myocardial infarction (TIMI) criteria (16).

### Myocardial Perfusion Scintigraphy

#### Patient Preparation

Patients were instructed to refrain from caffeine and methylxanthine-containing substances and to avoid nitrates, calcium channel blockers, and beta-blockers for at least 24 h before the scan. All scans were performed after overnight fasting.

#### MPS Acquisition Protocol

Each patient underwent rest-stress CZT SPECT (Discovery NM/CT 570c; GE Healthcare, Haifa, Israel) imaging according to a single-day protocol (mean 8 ± 2 days from ICA). All patients were imaged in the supine position with arms placed over their heads. Before the first dynamic acquisition, a low-dose CT scan (tube voltage 120 kV, tube current 20 mA, rotation time 0.8 s, helical pitch 0.969:1, slice thickness 5 mm, and interstice interval of 5 mm) was performed for heart positioning and for attenuation correction (AC).

Three MBq/kg of ^99m^Tc-Sestamibi were injected at rest using a syringe pump intravenously as a 5 ml bolus (injection rate 1 ml/sec) followed by saline flush (20 ml with the rate 2 ml/s, using an automatic injector Ulrich Missouri XD 2001 Ulrich GmbH & Co. KG, Ulm Germany). List mode ECG-gated dynamic data acquisition started 10 s prior to the radiopharmaceutical bolus injection and acquired for 610 s. After 40 min from rest tracer injection, a 7 min long standard ECG-gated (16 framed per cardiac cycle) rest acquisition was performed using a dedicated patient positioning application in order to obtain the same coordinates of the heart as in the previous scan. The stress study was performed immediately after.

After 2 min of intravenous infusion of adenosine (160 mcg/kg/min), the second dose of ^99m^Tc Sestamibi (9 MBq/kg) was injected, and list mode dynamic data acquisition of 610 s was started 10 s prior to the radiotracer injection. The infusion of adenosine continued for additional 2 min (17). After that, as for the rest scan, patients were removed from the gamma-camera and a stress standard ECG-gated scan was acquired after 45 min from the tracer injection.

#### Conventional CZT Data Reconstruction

Low dose CT scans were transferred to the Xeleris workstation to obtain AC maps. The alignment of perfusion and CT data was done with visual control. CZT images were reconstructed on the dedicated workstation (Xeleris 4.0; GE Healthcare, Haifa, Israel) using maximum-penalized-likelihood iterative reconstruction (60 iterations; Green OSL Alpha 0.7; Green OSL Beta 0.3) to acquire perfusion images in standard cardiac axes (short axis, vertical long axis, and horizontal long axis). The software Myovation for Alcyone (GE Healthcare, Haifa, Israel) was used for image reconstruction, and the Butterworth post-processing filter (frequency 0.37; order 7) was applied to the reconstructed slices. The reconstruction was performed in a 70 × 70 pixels matrix with 50 slices.

Raw MPS-CZT data at stress and at rest were visually analyzed for motion and attenuation artifacts. Stress/rest images were analyzed with a commercially available software package Corridor 4DM (University of Michigan, Ann Arbor, MI, USA) on AC images.

Each of 17 segments was scored based on semiquantitative 5-point scoring system (from 0 = normal uptake to 4 = absent radiotracer distribution) (18). Accordingly, the sum of the stress scores of all segments (SSS) and the sum of the rest scores of all segments (SRS) was quantified. A summed difference score (SDS) was calculated as the difference between SSS and SRS. Image processing was performed at the Core Facility “Medical Genomics” (Tomsk National Research Medical Center, Tomsk, Russia).

#### Analysis of Gated Images

LV functional analysis was performed from 16-frames reformatted images using commercially available software (Corridor4DM, Invia, Ann Arbor, MI).

#### Dynamic CZT Data Analysis

Dynamic CZT imaging was processed as previously published (19). In brief, the acquired data were initially reconstructed (in 70 × 70 pixels matrix; 50 slices) and re-binned into 20 frames: 12 frames of 10 s each and 8 frames of 30 s each. The reframed and corrected (for stress dataset) dynamic images were reconstructed using penalized maximum likelihood expectation maximization iterative algorithm. Finally, the reconstructed dynamic images, as well as CT AC maps, were processed by 4DM Reserve (2015 version) application. The time–activity curves (TAC) for the input function and whole LV myocardium as well as for the left anterior descending coronary artery (LAD), left circumflex coronary artery (LCx) and right coronary artery (RCA) vessel territories were generated semi-automatically. The region of interest (ROI) for input function was located on the valve plane and included parts of the LV cavity and left atrium. Manual motion correction of the dynamic dataset was performed in accordance with manufacturer's recommendation (20). The myocardial tracer uptake was estimated using a 1 tissue compartment model (1TCM) (21). To convert the tracer uptake rate to MBF values the Renkin–Crone flow model was used with the following parameters: α = 0.879, β = 0.337 for AC and α = 0.814, β = 0.065 for non-attenuation corrected (NAC) images (20–23). The value of MFR was calculated as the MBF ratio (MBF stress/MBF rest). Additionally, the absolute difference between stress MBF and rest MBF as flow difference (FD) was calculated (24, 25). The calculation of quantitative parameters was performed on both AC and NAC images.

### Cardiac Magnetic Resonance Acquisition and Image Analysis

Patients underwent CMR imaging 7 ± 2 days after admission, using a 1.5-Tesla clinical magnetic resonance scanner (Vantage Titan, Toshiba, Japan). Images were acquired during multiple breath-holds with a cardiac software package. ECG-gated cine images were obtained for functional analysis using a steady-state free precession sequence (slice thickness 8 mm, slice gap 2 mm, average repetition time (TR) and echo time (TE) 3.7/1.9 ms, respectively, flip angle 72°, FOV 360 × 360 mm, matrix 240 × 128, typically 12 phases per cardiac cycle), in the following orientations: two-chamber long axis, four-chamber long axis, and a short axis stack covering LV from the base to apex (typically 10 slices).

Prior to contrast administration, breath-hold T2-weighted spin-echo images were acquired to visualize infarct-related edema and intramyocardial hemorrhage (IMH) in short-axis orientation covering the whole LV. Typical parameters were TR = 1,774 ms; TE 80 ms.

Late gadolinium enhancement imaging was performed using an inversion recovery gradient echo sequence 8–15 min after administration of 0.15–0.2 mmol/kg gadolinium-based contrast agent Gadobutrol (Gadovist, Bayer Healthcare, Germany) in short-axis orientation covering the whole LV. Typical parameters were the following: slice thickness 7–10 mm; TR 9.1 mm; TE 3.6 ms; flip angle 17°; and TI 240–360 ms nulled to normal myocardium.

Myocardial edema (ME) was defined as an area with a high signal (hyperintensive) and was manually delineated and quantified. IMH was defined as an area with a low (attenuated) signal within the edematous myocardium and was manually delineated and quantified.

Infarct size, MVO, and myocardial edema were expressed as % of LV mass. Infarct size was quantified in a contrast-enhanced T1-WI,10 min post contrast administration using automatic algorithm EWA (Expectation Maximization, weighted intensity, a priori information) (26) as hyper enhanced areas whereas MVO was identified as a region of hypoenhancement within the region of the hyperenhanced infarct. Myocardial edema was quantitatively assessed on T2-weighted images. All image analysis was done using the freely available software Segment version 2.2 R6589 (27).

### Statistical Analysis

Data were assessed for normal distribution using the Shapiro–Wilk test. Continuous variables were expressed as mean ± standard deviation (SD) or as median with interquartile range (IQR, Q25–Q75). Differences between independent groups were determined by the Mann–Whitney U test. Spearman test was used to estimate the correlation coefficient between quantitative variables. Categorical variables were compared using Fisher's exact test. Survival analysis was performed using the Kaplan–Meier method. *p* < 0.05 was considered statistically significant. Statistical analyses were performed using STATISTICA 10.0 (StatSoft Inc, Tusla, OK, USA) and MedCalc version 18.9.1 (MedCalc Software, Mariakerke, Belgium).

## Results

### Patients

**Figure 1:**
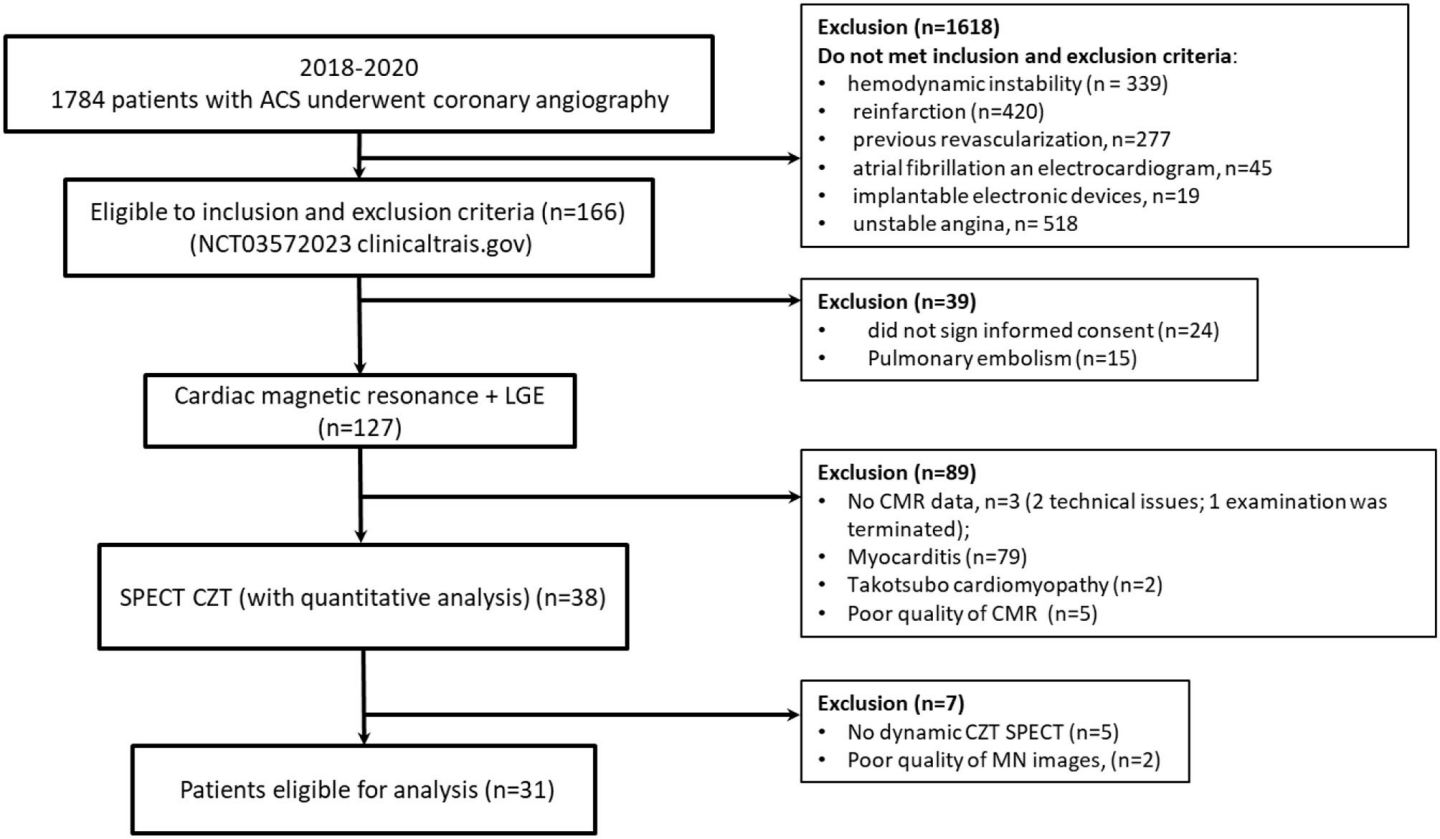
Flow diagram showing the patients included in the study. MINOCA, Myocardial infarction with non-obstructive coronary arteries; MICAD, Myocardial infarction with obstructive coronary artery disease; ACS, acute coronary syndrome.

The flow chart of the study is presented in Figure 1. A total of 31 patients were enrolled in the study. Detailed clinical characteristics of the study group are presented in Table 1. Based on ICA data 10 (32%) patients with AMI had non-obstructive coronary artery and 21 (68) obstructive coronary artery lesions. Most of the patients had hypertension and dyslipidemia. The length of hospital stay was 11 ± 2 days and was similar between MINOCA and MICAD. In 1 year follow-up 2(9.5%) of patients with MICAD had MI, no MACE was observed in MINOCA (Supplementary Figure S1).

**Table 1:** The clinical characteristics of patients.

	**All patients (*n* = 31)**	**MINOCA (*n* = 10)**	**MICAD (*n* = 21)**	**p MINOCA vs. MICAD**
Men, *n* (%)	19 (61.3)	5 (50.0)	14 (66.6)	0.03
Age, Me (Q25; Q75)	62 (56; 70)	68 (57; 79)	62 (56;68)	0.35
Hypertension, *n* (%)	24 (77.4)	10 (100)	14 (66.6)	0.61
Dyslipidemia, *n* (%)	25 (80.6)	10 (100)	15 (71.4)	0.08
Overweight, *n* (%)	12 (38.7)	3 (30.0)	9 (42.8)	0.13
Family history of CAD, *n* (%)	18 (58.1)	6 (60.0)	12 (57.1)	0.64
Smoking, *n* (%)	13 (41.9)	3 (30.0)	10 (47.6)	0.36
Diabetes mellitus, *n* (%)	4 (12.9)	0	4 (19.0)	0.06
GFR, ml / min / 1.73 m^2^, Me (Q25; Q75)	72.0 (54.0; 89.0)	64.5 (53.0; 72.0)	77.0 (65.0; 92.0)	0.7
History of angina pectoris, *n* (%)	12 (38.7)	6 (60.0)	6 (28.6)	0.78
A history of stroke, *n* (%)	2 (6.5)	0	2 (9.5)	0.27
Peripheral atherosclerosis, n (%)	9 (29.0)	3 (30.0)	6 (28.6)	0.41
Time of admission to the hospital, min, Me (Q25; Q75)	226 (120; 465)	600 (120.0; 1,080.0)	180 (98; 236.0)	0.01
STEMI, *n* (%)	23 (74.2)	6 (60.0)	17 (80.9)	0.03
GRACE, risk, Me (Q25; Q75)	2.0 (2.0; 5.0)	2.0 (2.0; 4.0)	2.2 (2.0; 5.0)	0.60
Thrombolytic therapy, *n* (%)	12 (38.7)	1 (10.0)	11 (52.4)	0.01
TIMI 2 flow, *n* (%)	6 (19.4)	5 (50.0)	1 (4.8)	0.05
Troponin I, ng/ml, 2 day	3.5 (0.6; 10.7)	0.5 (0.1;3.3)	8.3 (2.0; 18.6)	0.004
Troponin I, ng/ml, 4 day	0.7 (0.3; 3.0)	0.4 (0.04; 0.9)	1.4 (0.5; 3.7)	0.04
Troponin I, ng/ml, 7 day	0.2 (0.1; 0.6)	0.06 (0.01; 0.2)	0.3 (0.2; 0.7)	0.0009
Wall motion score index	1.1 (1.0; 1.5)	1.0 (1.0; 1.5)	1.2 (1.1; 1.5)	0.04
Left ventricular ejection fraction,%	63.0 (53.0; 66.0)	66.5 (64.0; 69.0)	57.0 (55.0; 65.0)	0.01
Length of hospital stay, days	11 ± 2	11 ± 2	11 ± 2	0.9

*All data are presented as mean ± SD. Median and interquartile range (IQR) and n (%); GFR, glomerular filtration rate; STEMI, ST-segment elevated myocardial infarction; TIMI, thrombolysis in myocardial infarction; GRACE, global registry of acute coronary events*.

### ICA Results

All patients underwent ICA within 1 day after admission to the emergency department. Mean time of ICA from the onset of symptoms was 315 ± 167 min. Per-patient analysis showed one-vessel disease in 14 (45%) patients, two vessel and three vessel disease in six (19%) and one (3%) patients, respectively. 13 (43%) patients had ≥50% stenosis in LAD, 4(13%) in LCx and 11(35%) in RCA. At per-vessel analysis, a total of 93 vessels were analyzed. Obstructive lesion was detected in 29 (31%) vessels −14(15%) in LAD, LCX in 4(4%) and 11(12%) in RCA. There were no obstructive lesions in the LMA. Infarct-related artery was LAD, LXC, and RAC in 12, 1, and 8 cases, respectively.

TIMI flow grade of 3 was observed in all patients with MINOCA and they were not stented. A total of 16/21 (76%) patients with MICAD underwent PCI with drug-eluting stenting of infarct-related arteries (IRA); in 3/16 cases, two stents were installed. In 13 IRA, TIMI flow grade before and after stenting was 3; in 5–0 and 3; in 1–0 and 2, respectively.

In four cases, a distal lesion of the IRA was detected (TIMI 0). After mechanical recanalization and balloon angioplasty, there was no blood flow in the distal segment of IRA (TIMI 0); PCI was not performed. In one case, non-occlusive thrombosis of the anterior descending artery was detected (TIMI2). Thromboaspiration was performed with the appointment glycoprotein IIb/IIIa receptor inhibitors (Eptifibatide). According to optical coherence tomography, performed the next day a hemodynamically insignificant plaque of 25% (TIMI 3) with areas of erosion was found, and a non-invasive management strategy was chosen.

### Cardiac Magnetic Resonance Results

**Figure 2:**
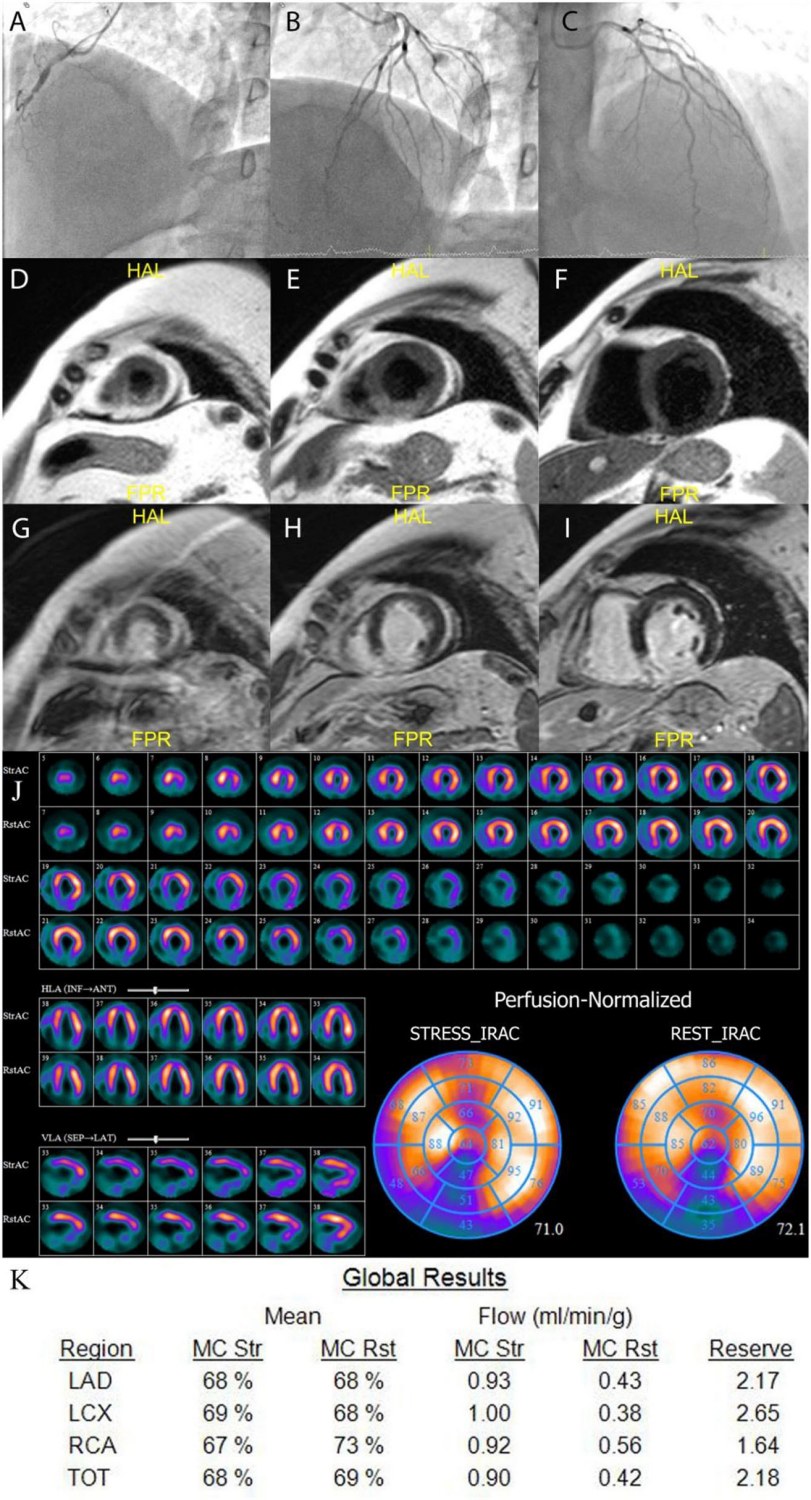
Example of MICAD patient. A 55 years old male with RCA occlusion **(A)** and no CAD in LAD and LCx **(B,C)**. CMR results: T2-weighted images **(D–F)** show transmural edema in the posterior and posterior-septal segments; late gadolinium enhancement images **(G–I)** show high signal intensity in the same regions indicating necrosis in the posterior and posterior-septal segments. The qualitative MPS **(J)** shows severe fixed perfusion defect in posterior wall and small reversible defect in mid and basal anterior wall. The quantitative CZT SPECT results **(K)** show abnormal MFR in RCA territory. LAD, left anterior descending artery; LCx, left circumflex artery; RCA, right coronary artery; TOT, total value; MC, motion correction; mL/min/g, milliliters per minute per gram; Rst, rest; Str, stress.

**Figure 3:**
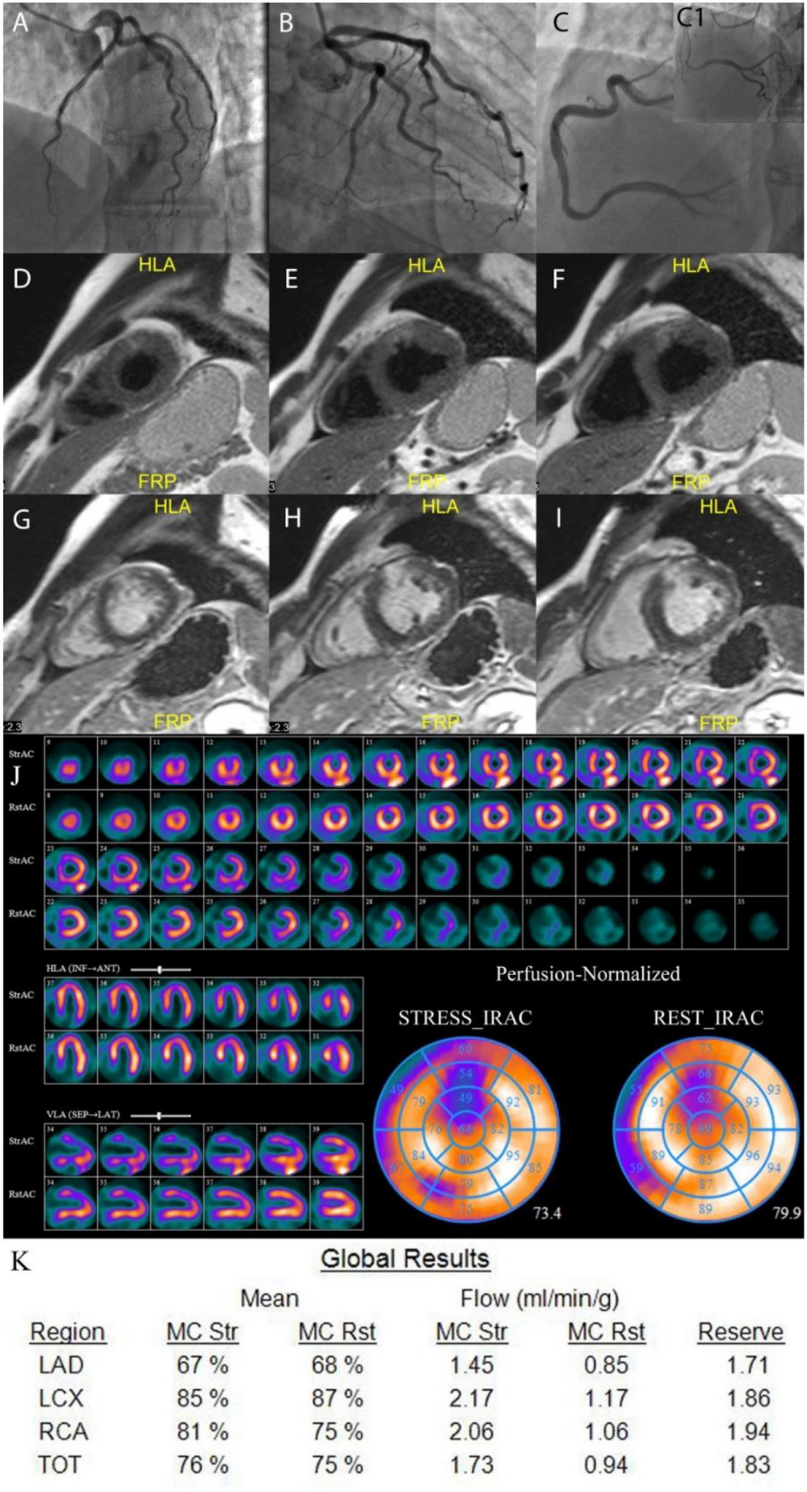
Example of MINOCA patient. A 46 years old male with no obstructive coronary arteries **(A–C1)**. CMR results: T2-weighted images **(D–F)** show transmural edema in the anterior segments; late gadolinium enhancement images **(G–I)** show high signal intensity in the same regions indicating necrosis in the anterior segments. The qualitative MPS **(J)** shows severe moderate to severe partially reversible perfusion defect in the anterior wall. The quantitative CZT SPECT results **(K)** show abnormal stress MBF and MFR in LAD vessel territory. LAD, left anterior descending artery; LCx, left circumflex artery; RCA, right coronary artery; TOT, total value; MC, motion correction; mL/min/g, milliliters per minute per gram; Rst, rest; Str, stress.

Based on all patient's sample analyses, late gadolinium enhancement, MVO, and myocardial edema were observed in 28 (90%), 11 (35%), and 24 (77%) patients, respectively. Interestingly, there were no MVO in patients with MINOCA. Quantitative values of infarct size, MVO, and myocardial edema are presented in Table 2. In patients with MICAD, the values of infarct size, MVO, and myocardial edema were significantly (<0.05) higher as compared to those in MINOCA. The combination of myocardial edema and late gadolinium enhancement was considered AMI (28, 29). A total of 93 vessel territories were analyzed. There were 39 (42%) vessel territories considered as having acute myocardial injury [5 (5%) in MINOCA and 34 (37%) in MICAD] and 54 (58%) with no myocardial injury [25 (27%) in MINOCA and 29 (31%) in MICAD]. The number of vessel territories with acute myocardial injury in MICAD subjects was significantly higher as compared to MINOCA (34 *vs*. 5, *p* = 0.005) (Supplementary Figure S2). Additional data describing the regional CMR analysis results is provided in the supplementary material (Supplementary Figure S3). Representative cases of patients MINOCA and MICAD are presented in Figures 2, 3.

**Table 2:** CMR result.

	**All patients**	**MINOCA**	**MICAD**	**p MINOCA vs. MICAD**
Infarct size (LGE, % of LV mass)	10.6 (1.9; 22.6)	1.8(0.0; 6.9)	19.4(10.4; 29.7)	0.0003
MVO, (% of LV mass)	0.0(0; 0.2)	0.0(0.0; 0.0)	0.1(0.0; 0.7)	0.01
Myocardial edema, (% of LV mass)	15(3.5; 24)	3.0(0.0; 12.0)	19.5(12.0; 30.0)	0.002

*All data are presented as median and interquartile range (IQR). LGE, late gadolinium enhancement; MVO, microvascular obstruction; LV, left ventricle*.

### MPS Results

In a mean of 8 ± 2 (min 4; max 13) days after admission a total of 20 patients underwent 2-days rest-stress dynamic SPECT protocol and 11 patients had a rest-stress single-day protocol. The results of MPS are presented in Table 3. A total of 25(81%) patients had abnormal (SSS>3) myocardial perfusion−7 (70%) in MINOCA and 21(100%) in MICAD. Patients with MICAD showed high values of SSS and SRS (*p* <0.01) and lower values of global sMBF, MFR, and FD when compared to the MINOCA group. Moreover, the values of stress EF and PER were significantly (*p* < 0.05) lower in MICAD *v*s. the MICAD subgroup.

**Table 3:** Myocardial perfusion scintigraphy results.

**Gated MPS and blood flow parameters**	**All pts with AMI (*n* = 31)**	**MINOCA (*n* = 10)**	**MICAD (*n* = 21)**	**p MINOCA vs. MICAD**
***MPS parameters***				
SSS (IQR)	6.0 (5.0; 12.0)	5.0 (3.0; 5.0)	9.0 (5.0; 13.0)	0.0025
SRS	4.0 (2.0; 7.0)	2.0 (1.0; 3.0)	6.0 (3.0; 11.0)	0.0029
SDS	3.0 (1.0; 5.0)	2.5 (1.0; 4.0)	4.0 (2.0; 5.0)	0.18
***MPS functional***				
Stress EDV, ml	127.0 (100.0; 162.0)	114.0 (107.0; 131.0)	150.0 (100.0; 177.0)	0.19
Stress ESV, ml	60. (32.0; 70.0)	45.0 (32.0; 47.0)	66.0 (36.0; 94.0)	0.049
Stress EF, %	58.0 (48.0; 66.0)	62.0 (58.0; 71.0)	53.0 (47.0; 60.0)	0.027
Rest EDV, ml	126.0 (94.0; 158.0)	111.5 (94.0; 127.0)	134.0 (94.0; 162.0)	0.16
Rest ESV, ml	51.0 (37.0; 67.0)	38.0 (35.0; 45.0)	54.0 (42.0; 89.0)	0.06
Rest EF, %	60.0 (50.0; 65.0)	63.0 (60.0; 66.0)	58.0 (49.0; 63.0)	0.036
***Absolute parameters with AC***				
Stress MBF, ml/min/g	1.04 (0.89; 2.02)	2.03 (1.73; 2.18)	0.97 (0.8; 1.04)	0.0005
Rest MBF, ml/min/g	0.77 (0.54; 1.24)	0.91 (0.57; 1.52)	0.75 (0.5; 1.15)	0.37
MFR	1.55 (1.04; 2.07)	1.96 (1.33; 3.31)	1.28 (0.98; 1.86)	0.048
FR, ml/min/g	0.35 (0.07; 0.76)	0.77 (0.5; 1.5)	0.16 (−0.02; 0.36)	0.02
***Absolute parameters with NAC***				
Stress MBF, ml/min/g	1.0 (0.7; 1.71)	2.02(1.71; 2.37)	0.86(0.72; 1.02)	0.00003
Rest MBF, ml/min/g	0.55(0.43; 0.77)	0.68(0.66; 0.86)	0.49(0.4; 0.57)	0.021
MFR	1.79(1.4; 2.44)	2.61(2.23; 3.14)	1.67(1.1; 1.9)	0.002
FD, ml/min/g	0.42(0.15; 079)	1.23(0.79; 1.69)	0.32(0.04; 047)	0.00001

*All data are presented as median and interquartile range (IQR).*

*AC, attenuation correction; NAC, non-attenuation correction; MPS, myocardial perfusion scintigraphy; SSS, summed stress score; SRS, summed rest score; SDS, summed difference score; EDV, end diastolic volume; ESV, end systolic volume; EF, ejection fraction; MBF, myocardial blood flow; mL/min-1/g-1, milliliters per minute per gram; MFR, myocardial flow reserve; FD, flow difference*.

Among 3 (10%) patients with normal myocardial perfusion 1 (3%) had abnormal CMR. The correlations between CZT-derived global quantitative flow values with infarct size, MVO, and myocardial edema derived by CMR are presented in Table 4. Significant (*p* < 0.05) correlation between infarct size and stress MBF was revealed. Moreover, NAC rest MBF demonstrated a significant correlation with IS, MVO, and ME. The weakest correlation was observed between CZT SPECT-derived absolute flow indexes and the extent of microvascular obstruction.

**Table 4:** The Spearman correlations between CMR and absolute MPS parameters.

	**IS**		**MVO**		**ME**	
***Absolute MPS parameters with AC***	**ρ**	***p*-value**	**ρ**	***p*-value**	**ρ**	***p*-value**
Stress MBF, ml/min/g	−0.68	<0.001	−0.50	0.0055	−0.58	0.0013
Rest MBF, ml/min/g	−0.11	0.5650	−0.08	0.6839	−0.14	0.4717
MFR	−0.48	0.0086	−0.30	0.1189	−0.39	0.0412
FR, ml/min/g	−0.53	0.0030	−0.32	0.0867	−0.43	0.0239
***Absolute MPS parameters with NAC***						
Stress MBF, ml/min/g	−0.77	<0.001	−0.53	0.0037	−0.70	<0.001
Rest MBF, ml/min/g	−0.58	0.001	−0.59	<0.001	−0.60	<0.001
MFR	−0.54	0.003	−0.30	0.1230	−0.54	0.0033
FD, ml/min/g	−0.65	<0.001	−0.46	0.0144	−0.61	<0.001

*IS, infarct size; MVO, microvascular obstruction; ME, myocardial edema; AC, attenuation correction; NAC, non-attenuation correction; MPS, myocardial perfusion scintigraphy; MBF, myocardial blood flow; mL/min-1/g-1, milliliters per minute per gram; MFR, myocardial flow reserve; FD, flow difference; ρ, Spearman's rho*.

### Relationship Between Cardiac Serum Troponin and MPS Results

According to the whole same analysis cTnI at 24 h correlated significantly (*p* < 0.05) with stress MBF and FD (both with AC and NAC). cTnI on day 4 correlated significantly with MFR_AC and FD_AC. cTnI level at 7 day showed a negative correlation (*p* < 0.01) with stress MBF_NAC and FD_NAC. Detailed correlations analyses of cardiac serum troponin with quantitative and visual MPS parameters are provided in Supplementary Tables S1–S3, Supplementary Figure S4 shows scatter plots and correlations between serial cTnI levels and MBF. Subgroup analysis showed a significant correlation only between cTnI level at 4 day and SRS (*p* = 0.03), cTnI level at 7 day, and SSS (*p* = 0.005) in patients with MINOCA. No significant correlation was found between cTnI and MPS values in the MICAD subgroup.

### MPS Regional Analysis

Regional analysis of CZT SPECT data was based on CMR findings. The mean interval between CMR and MPS was 3 days (min 0; max 5). Vessel territories with a CMR pattern of acute myocardial injury (LGE plus ME) were considered as CMR positive and ones with no such signs–as CMR negative. The result of the regional analysis for the whole sample is provided in Table 5. sMBF, MFR and FD were significantly (<0.01) lower in vessel territories considered CMR positive compared to those without myocardial injury. Subgroup analysis showed that only in MINOCA subjects, sMBF_AC, MFR_AC, and FD_AC were significantly (<0.05) lower in CMR positive vessel territories, as compared to CMR negative ones. Detailed data is provided in Supplementary Table S4.

**Table 5:** CMR based regional analysis of SPECT CZT data.

	**Vessel territories with no acute myocardial injury (*n* = 54)**	**Vessel territories with acute myocardial injury (*n* = 39)**	**P. Mann-whitney U test**
***Absolute MPS parameters with AC***			
Stress MBF, ml/min/g	1.85 (1.17; 2.28)	1.12 (0.93; 1.74)	0.000095
Rest MBF, ml/min/g	0.94 (0.66; 1.41)	0.98 (0.61; 1.63)	0.95 (NS)
MFR	1.67 (1.17; 2.36)	1.15 (0.88; 1.88)	0.0003
FD, ml/min/g	0.52 (0.2; 1.22)	0.16 (−0.15; 0.5)	0.0002
***Absolute MPS parameters with NAC***			
Stress MBF, ml/min/g	1.33 (0.94; 2.08)	0.98 (0.73; 1.79)	0.0075
Rest MBF, ml/min/g	0.71 (0.57; 0.88)	0.58 (0.43; 0.9)	0.13 (NS)
MFR	2.0 (1.53; 2.89)	1.64 (1.0; 2.36)	0.033
FD, ml/min/g	0.56 (0.36; 1.32)	0.33(0.05; 0.57)	0.003

*All data are presented as median and interquartile range (IQR). CMR, cardiac magnetic resonance; MPS, myocardial perfusion scintigraphy; MBF, myocardial blood flow; MFR, myocardial flow reserve; FD, flow difference; mL/min-1/g-1, milliliters per minute per gram; AC, attenuation correction; NAC, non-attenuation correction*.

## Discussion

This study showed that in patients with AMI the values of both visual and quantitative MPS indexes correlate significantly with the level of high-sensitivity serum cardiac troponin I and CMR findings. In particular, the values of regional sMBF, MFR, and FD are significantly decreased in LV territories characterized by myocardial injury. Finally, the values of quantitative MPS are lower in MICAD compared to patients with MINOCA.

These results indicated that in patients with acute myocardial infarction, the impairment of flow and reserve, assessed by SPECT CZT, is correlated with extension and severity of myocardial edema and infarct size.

### Myocardial Blood Flow in Acute Coronary Syndrome

In line with the previous study by Stewart et al. where the comparison in rest and stress MBF (by PET with ^13^N-ammonia) were performed on the day 9 after PCI in infarct-related and reference artery (5), we found out significantly reduced MBF, MFR and FD (both AC and NAC) in LV territories with myocardial injury. The disturbances of myocardial microcirculation due to myocardial edema and injury may be considered as a reason for quite low values of global MBF both on stress and rest. In a previous study enrolled patients with ST-elevation myocardial infarction (STEMI), Pan et al. showed significantly lower rest MBF in infarcted territories compared to those of reference territories (30.5 ± 7.4 *vs*. 73.4 ± 8.1 ml/min/100 ml, *p* < 0.001) (30). Moreover, a moderate correlation between peak high-sensitivity troponin T and rest MBF was revealed by the authors. In our study, even though the majority of patients had STEMI, the rest of MBF neither correlates with cTnI nor differs in injured and non-injured myocardial regions. Moreover, the values of rest MBF in regions both with and without myocardial injury were higher compared to the study of Pan et al. (30).

### SPECT CZT Studies of MBF and MFR in Patients With Stable CAD

Although previous studies showed that SPECT MPS may provide diagnostic and prognostic information regarding cardiac events in high-risk patients (31), particularly in those with AMI, (32–34), this study elucidates the value of quantitative SPECT CZT in MINOCA and MICAD individuals. CZT SPECT derived MBF and MFR are currently well-validated against PET (8, 9, 11, 20), invasive angiography (19, 35–37) and invasive FFR (8, 38–40).

Previous CZT studies demonstrated the feasibility of CZT SPECT to detect differences in MBF and MFR between patients with and without stable CAD (11) and reduction of MBF and MFR according to the number of vessels with stenosis. It was shown that high-risk patients with stable CAD have reduced values of stress MBF and MFR (12, 19, 38). However, there is a paucity of data regarding the diagnostic value of CZT SPECT in patients with the acute coronary syndrome. Han et al. (41) reported significantly lower values of MFR in patients with a history of myocardial infarction (1.56 ± 0.26) in comparison to those without (2.71 ± 1.02). However, the use of different tracers (^201^Tl in the study of Han *vs*. ^99m^Tc in the others) and their different extraction fractions make these results difficult to compare.

To our knowledge, for the first time, we assessed the value of CZT in ACS patients and compared it to CMR findings. We found significantly reduced MBF and MFR in LV regions with myocardial injury, as detected by CMR. Our findings imply that the use of CZT SPECT can detect not only regional microcirculation abnormalities related to acute coronary syndrome (42, 43) but also characterized the severity of the injury, as indicated by the moderate negative correlation between MPS flow indexes and CMR variables.

Moreover, this study provides new insight into MBF and MFR in patients with AMI with and without obstructive coronary arteries. In fact, we found out that both visual and quantitative parameters of myocardial perfusion in the MINOCA subgroup were in the normal range or impaired mildly.

The prognosis in MINOCA compared to MICAD remains controversial. Although earlier studies suggested a better prognosis of MINOCA as compared to MI with obstructive coronary artery disease (44, 45) more recent studies showed no differences in prognosis among MINOCA and patients with MICAD (46–48). In this study, two patients with MICAD suffered myocardial infarction during the follow-up period.

When MINOCA was compared to stable CAD patients with no obstructive arteries, it was revealed worse 1 year prognosis in terms of mortality, myocardial infarction, and revascularization in MINOCA (49). In our study, MINOCA patients showed abnormal myocardial perfusion, despite smaller than the one of MICAD group, that may be associated with worse prognosis (50, 51). The usefulness of quantitative SPECT in patient with ACS, whether with or without obstructive CAD, may indicate the myocardium at risk as well as residual ischemia in infarct-related artery territory, which may have prognostic value and determine of treatment strategy (52).

### Limitation

Few patients limits the statistical power of this study, as well as the one-center enrolment and observation design. Nevertheless, we found statistically significant differences in regional values of SPECT CZT-derived quantitative flow measures between LV territories with and with no myocardial injury as well as the correlation with cTnI. The measurement of MBF and MFR as well as CMR were performed 7–8 days past PCI. In the MICAD group we performed MPS and MRI after stenting, which may affect the results of a visual and quantitative assessment of myocardial perfusion, flow, and injury. On the other hand, the time interval between MPS, MRI, and cTnI (on day 7) did not exceed 1 day, so the association between findings was not affected by the different times of image acquisition.

## Conclusion

The results of this study demonstrated the feasibility of CZT SPECT to assess global and regional abnormalities of MBF and MFR in AMI subjects. On the global level, SPECT CZT-derived flow measures were associated with high sensitivity troponin I as well as the extent of edema, MVO, and infarct size detected by CMR. On the regional level, quantitative SPECT CZT measures were significantly lower in vessel territories with myocardial injury. Since MINOCA patients are characterized by mild reduction of myocardial blood flow and perfusion assessed visually and quantitatively, it implies that this group of patients has a more pronounced risk of cardiac events and needs more aggressive observation and treatment, despite the absence of obstructive coronary artery lesion and TIMI grade 3 flow. It requires further large-scale studies to test the prognostic significance of SPECT-derived MBF and MFR in patients with the acute coronary syndrome.

## Data Availability Statement

The raw data supporting the conclusions of this article will be made available by the authors, without undue reservation.

## Ethics Statement

The studies involving human participants were reviewed and approved by Committee on Biomedical Ethics of the Cardiology Research Institute, Tomsk National Research Medical Center, Russian Academy of Sciences. The patients/participants provided their written informed consent to participate in this study.

## Author Contributions

KZ and AG: conception of the design, drafting of the manuscript, data analysis, and final approval. DV: clinical data analysis and revising the manuscript. OM: CMR data analysis and revising the manuscript. AVM, ANM, and MG: nuclear data processing, analysis, and revising the manuscript. AB: invasive coronary angiography data analysis and revising the manuscript. VR: conception of the idea, revising the manuscript, and final approval. All authors contributed to the article and approved the submitted version.

## Conflict of Interest

The authors declare that the research was conducted in the absence of any commercial or financial relationships that could be construed as a potential conflict of interest.

## Publisher's Note

All claims expressed in this article are solely those of the authors and do not necessarily represent those of their affiliated organizations, or those of the publisher, the editors and the reviewers. Any product that may be evaluated in this article, or claim that may be made by its manufacturer, is not guaranteed or endorsed by the publisher.
