# Supplementary material for: Myocardial Blood Flow and Flow Reserve in Patients With Acute Myocardial Infarction and Obstructive and Non-Obstructive Coronary Arteries: CZT SPECT Study
Source: Front Nucl Med. 2022 Jul 6;2:935539. doi: 10.3389/fnume.2022.935539 (PMC11440855; doi:10.3389/fnume.2022.935539)
Supplement: Supplementary file 1 [file Data_Sheet_1.docx]

**Supplementary material**

| **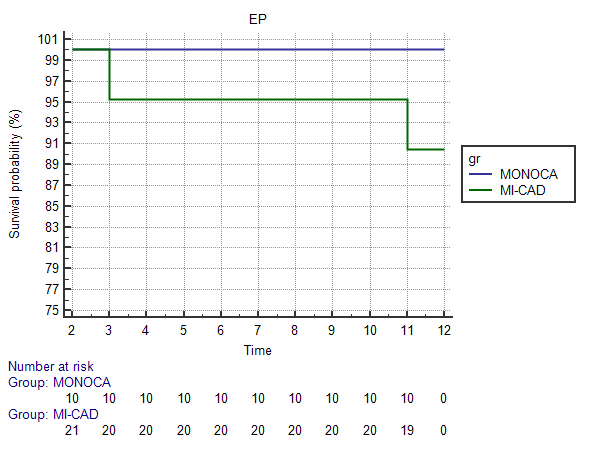** |
| --- |
| Fig1s. Kaplan-Mayer analysis showed that MICAD group exhibits not-significant tendency (p= 0,32) to more frequent MACE as compared to MINOCA. EP, composite end point (all-cause death, myocardial infarction, unstable angina, revascularization). |

| **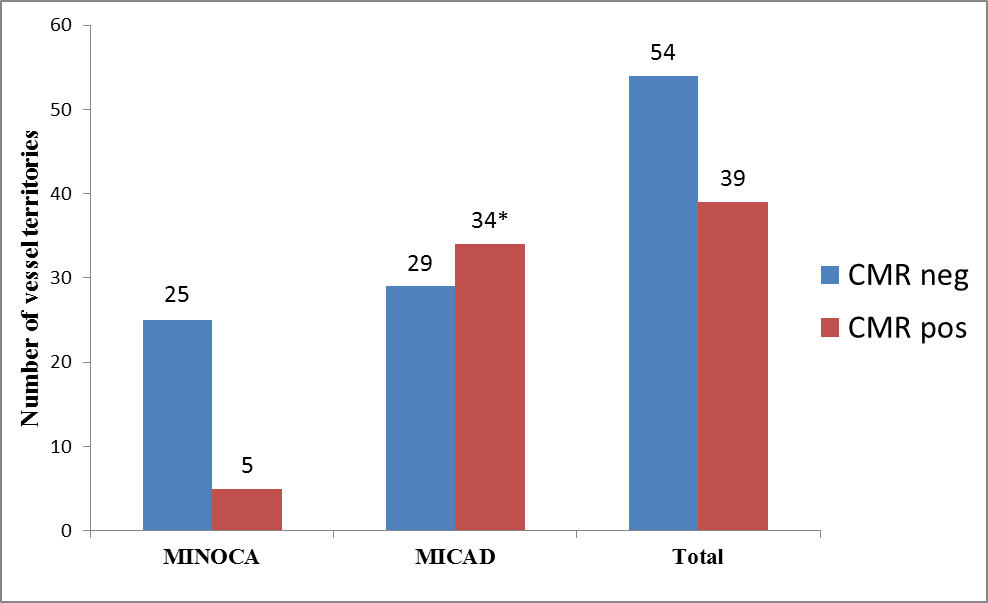** |
| --- |
| Fig2s. The number of CMR positive in CMR negative vessel territories in MINOCA and MICAD patients. * - the number of CMR positive vessel territories in MICAD subjects was significantly (p=0.005 by Fisher exact test) higher as compared to MINOCA. |

| **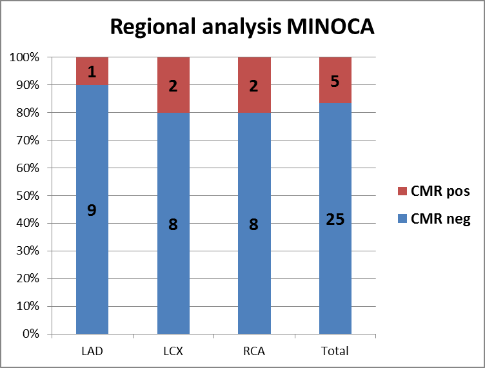** | **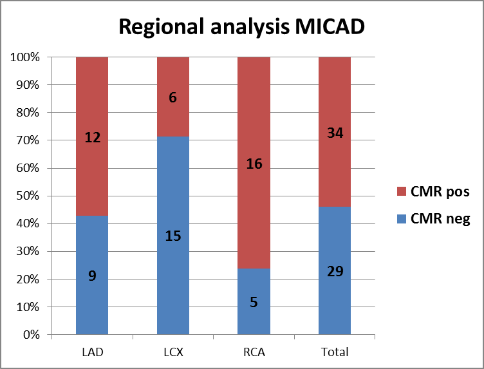** | **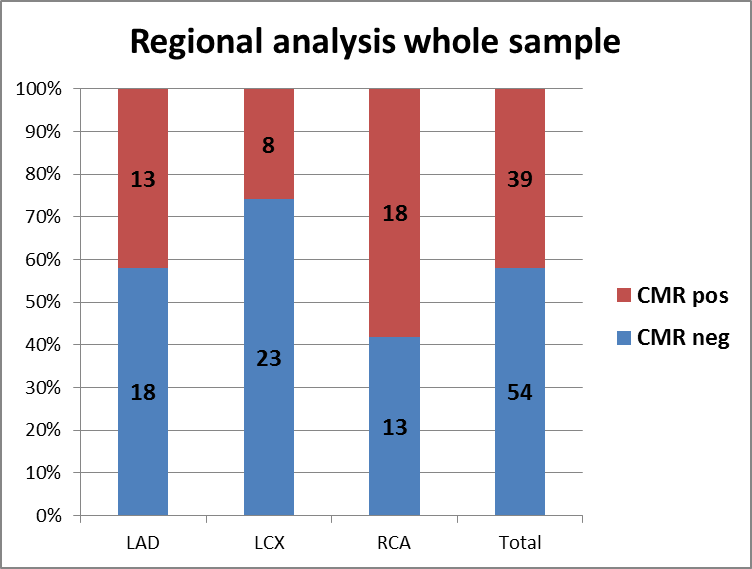** |
| --- | --- | --- |
| A | B | C |
| Fig 3s. Regional analysis of CMR positive and CMR negative vessel territories in MINOCA (A), MICAD (B) subgroup and whole sample (C). | | |

**Table 1s. The Spearman correlations between serial Troponin I measurements and quantitative MPS parameters with NAC**

|  | **sMBF_NC** | | **rMBF_NC** | | **MFR_NC** | | | **FD_NC** | | |  |
| --- | --- | --- | --- | --- | --- | --- | --- | --- | --- | --- | --- |
|  | ρ | p | ρ | p | | ρ | p | | ρ | p | |
| Troponin I, ng/ml, 2 day | -0.39 | 0.03* | -0.29 | 0.11 | | -0.27 | 0.14 | | -0.39 | 0.03* | |
| Troponin I, ng/ml, 4 day | -0.30 | 0.09 | -0.21 | 0.26 | | -0.23 | 0.21 | | -0.29 | 0.11 | |
| Troponin I, ng/ml, 7 day | -0.47 | 0.007* | -0.33 | 0.07 | | -0.34 | 0.05 | | -0.46 | 0.009* | |
| * p<0.05; ρ- Spearman correlations coefficients; MBF, myocardial blood flow; MFR, myocardial flow reserve; FD, flow difference; mL/min-1/g-1, milliliters per minute per gram; AC, attenuation correction; NAC, non-attenuation correction; | | | | | | | | | | | |

**Table 2s. The Spearman correlations between serial Troponin I measurements and quantitative MPS parameters with AC**

|  | **sMBF_AC** | | **rMBF_AC** | | **MFR_AC** | | **FD_AC** | |  |
| --- | --- | --- | --- | --- | --- | --- | --- | --- | --- |
|  | ρ | p | ρ | p | ρ | p | ρ | p |  |
| Troponin I, ng/ml, 2 day | -0.39 | 0.02* | 0.014 | 0.93 | -0.32 | 0.07 | -0.42 | 0.01* |  |
| Troponin I, ng/ml, 4 day | -0.24 | 0.17 | 0.13 | 0.47 | -0.39 | 0.02* | -0.43 | 0.01* |  |
| Troponin I, ng/ml, 7 day | -0.35 | 0.05 | -0.06 | 0.73 | -0.22 | 0.22 | -0.26 | 0.14 |  |
| * p<0.05; ρ- Spearman correlations coefficients; MBF, myocardial blood flow; MFR, myocardial flow reserve; FD, flow difference; mL/min-1/g-1, milliliters per minute per gram; AC, attenuation correction; NAC, non-attenuation correction; | | | | | | | | | |

**Table 3s. The Spearman correlations between serial Troponin I measurements and visual MPS parameters in the whole sample**

|  | **SSS** | | **SRS** | | **SDS** | |
| --- | --- | --- | --- | --- | --- | --- |
|  | ρ | **p value** | ρ | **p value** | ρ | **p value** |
| Troponin I, ng/ml, 2 day | 0,4 | 0,02* | 0,5 | 0,004* | 0,04 | 0,8 |
| Troponin I, ng/ml, 4 day | 0,39 | 0,02* | 0,37 | 0,03* | 0,13 | 0,45 |
| Troponin I, ng/ml, 7 day | 0,56 | 0,0008* | 0,4 | 0,02* | 0,34 | 0,05 |
| * p<0.05; ρ- Spearman correlations coefficients; SSS, summed stress score; SRS, summed rest score; SDS, summed difference score. | | | | | | |

**Table 4s. Regional (by vessel territories) myocardial perfusion scintigraphy results**

|  | **MINOCA (n=30)** | | | **MICAD (n=63)** | | |
| --- | --- | --- | --- | --- | --- | --- |
|  | **Myocardial injury (n=5)** | **No myocardial injury (n=25)** | **p (U test))** | **Myocardial injury (n=34)** | **No myocardial injury (n=29)** | **p (U test)** |
| ***Absolute parameters with NAC*** | | | | | | |
| Stress MBF, ml/min/g | 1.81(1.78;2.05)) | 2.19(1.69;3.35) | 0,23 | 0.97(0.73;1.15) | 0.98(0.85;1.26) | 0,48 |
| Rest MBF, ml/min/g | 0.9(0.58;1.02) | 0.78(0.66;0.9) | 0,92 | 0.58(0.41;0.9) | 0.6(0.43;0.84) | 0,81 |
| MFR | 1.74(1.7;3.1) | 2.88(2.16;3.97) | 0,28 | 1.43(0.94;2.24) | 1.64(1.24;2.0) | 0,53 |
| FD, ml/min/g | 0.76(0.31;1.23) | 1.43(0.71;2.51) | 0,12 | 0.23(-0.12;0.5) | 0.41(0.17;0.51) | 0,34 |
| ***Absolute parameters with AC*** | | | | | | |
| Stress MBF, ml/min/g | 1.87(1.45;1.92) | 2,19(1.91;2.76) | 0,036* | 1.04(0.91;1.64) | 1.19(1.06;1.72) | 0,093 |
| Rest MBF, ml/min/g | 1.64(0.85;2.5) | 1.02(0.72;1.46) | 0,384742 | 0.9(0.57;1.3) | 0.92(0.56;1.14) | 0,86 |
| MFR | 1.14(0.77;1.71) | 2.18(1.46;2.77) | 0,031* | 1.21(0.91;2.03) | 1.45(1.13;2.0) | 0,21 |
| FD, ml/min/g | 0.23(-0.58;0.55) | 1.0(0.54;1.71) | 0,013* | 0.16(-0.13;0.55) | 0.42(0.13;0.52) | 0,14 |
| All data are presented as median and interquartile range (IQR). * p<0.05; CMR, cardiac magnetic resonance; MBF, myocardial blood flow; MFR, myocardial flow reserve; FD, flow difference; mL/min-1/g-1, milliliters per minute per gram; AC, attenuation correction; NAC, non-attenuation correction; | | | | | | |

| 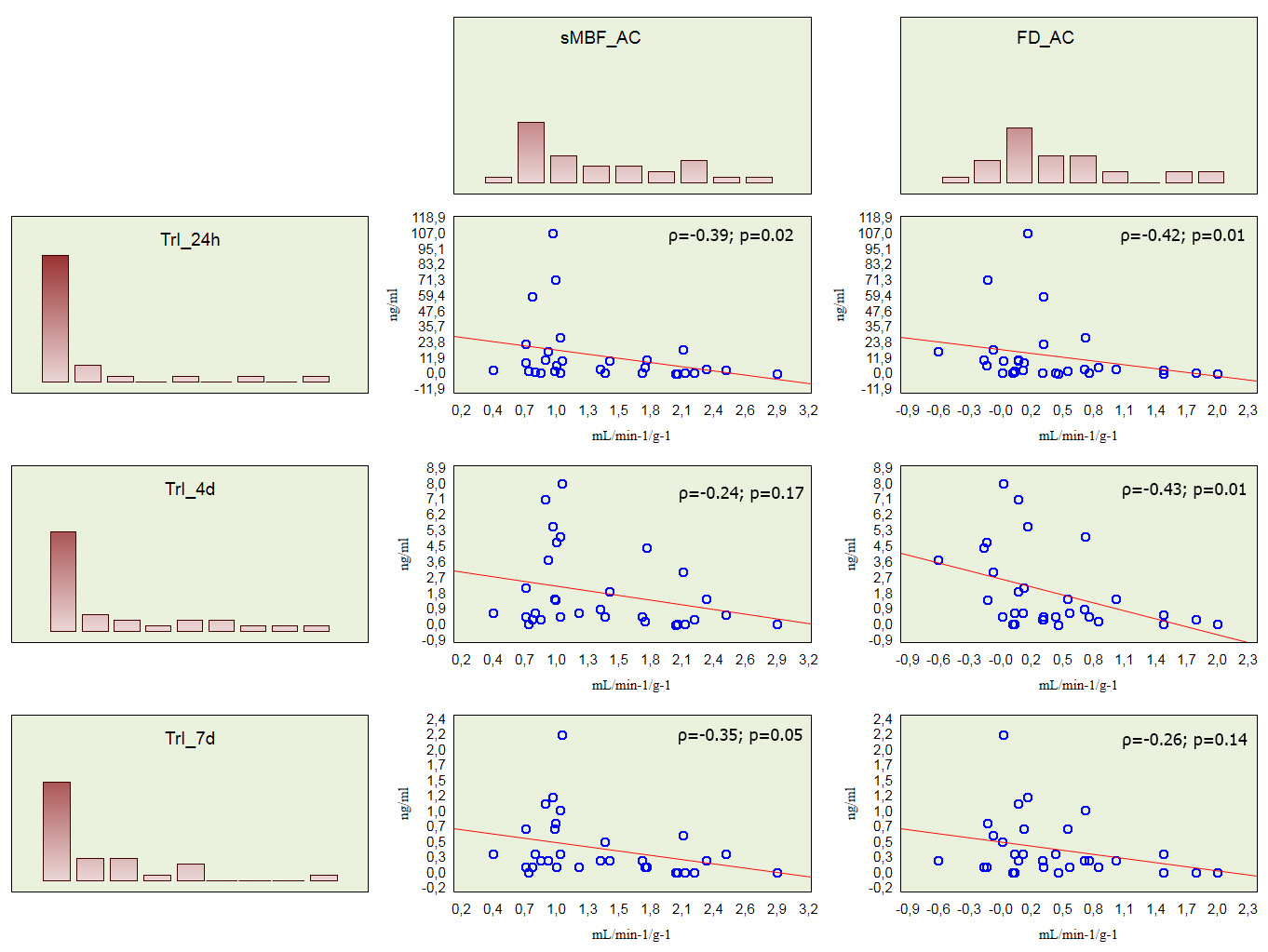 |
| --- |
| Fig3s. Correlations between Troponin I levels and MBF (with AC) for days 2, 4 and 7. AC, attenuation correction, sMBF, stress myocardial blood flow; FD, flow difference; TrI_24h, Troponin I day 2; TrI_4d, Troponin I day 4; TrI_7d, Troponin I day 7; mL/min-1/g-1, milliliters per minute per gram; ρ- Spearman correlations coefficients |
